# Transporting and implementing a caregiver-mediated intervention for toddlers with autism in Goa, India: evidence from the social ABCs

**DOI:** 10.3389/fresc.2024.1214009

**Published:** 2024-02-14

**Authors:** Jessica A. Brian, Erin M. Dowds, Kate Bernardi, Andre Velho, Mahera Kantawalla, Nandita de Souza

**Affiliations:** ^1^ Autism Research Centre, Bloorview Research Institute, Holland Bloorview Rehabilitation Hospital, Toronto, ON, Canada; ^2^ Department of Paediatrics, University of Toronto, Toronto, ON, Canada; ^3^ Sethu Centre for Child Development and Family Guidance, Goa, India

**Keywords:** autism, toddlers, early intervention, parent-mediated, virtual delivery, cultural adaptation, low-middle-income countries, transportability

## Abstract

**Introduction:**

Autism is a global health priority with an urgent need for evidence-based, resource-efficient, scalable supports that are feasible for implementation in low- and middle-income countries (LMICs). Initiating supports in the toddler years has potential to significantly impact child and family outcomes. The current paper describes the feasibility and outcomes associated with a Canadian-developed caregiver-mediated intervention for toddlers (the Social ABCs), delivered through a clinical service in Goa, India.

**Methods:**

Clinical staff at the Sethu Centre for Child Development and Family Guidance in Goa, India, were trained by the Canadian program development team and delivered the program to families seen through their clinic. Using a retrospective chart review, we gathered information about participating families and used a pre-post design to examine change over time.

**Results:**

Sixty-four families were enrolled (toddler mean age = 28.5 months; range: 19–35), of whom 55 (85.94%) completed the program. Video-coded data revealed that parents learned the strategies (implementation fidelity increased from *M* = 45.42% to 76.77%, *p* < .001, with over 90% of caregivers attaining at least 70% fidelity). Toddler responsivity to their caregivers (M = 7.00% vs. 46.58%) and initiations per minute (*M* = 1.16 vs. 3.49) increased significantly, *p*'s < .001. Parents also reported significant improvements in child behaviour/skills (*p* < .001), and a non-significant trend toward reduced parenting stress (*p* = .056).

**Discussion:**

Findings corroborate the emerging evidence supporting the use of caregiver-mediated models in LMICs, adding evidence that such supports can be provided in the very early years (i.e., under three years of age) when learning may be optimized.

## 1 Introduction

Autism has been identified as a global health priority, with an urgent need for high-quality, resource-efficient, scalable interventions and supports that are evidence-based and feasible for implementation in low- and middle-income countries (LMICs) (1). According to the Global Health report (2) 95% of people with autism live in developing countries. The World Health Organization (WHO)'s Global Strategy for Women's, Children's and Adolescents' Health and the United Nations Sustainable Development Goals highlight the importance of providing nurturing care to all children (3). Caregiver-mediated programs meet the call for scalable resource-efficient models that hold promise for implementation in LMICs (1, 4). Moreover, such programs value and promote caregivers' natural strengths, and empower them with new skills that can be used to foster their child's development (3, 5). Given the high need for effective and efficient interventions in LMICs, it has been argued that “parent-mediated interventions in ASD are not an optional extra or adjunct in this region [India] but imperative, vital, and very likely the mainstay of therapy for innumerable families with minimal or no access to resources” (6).

While most interventions have been developed in high-income countries [with only 5% having been developed in Africa, South America, or India (7)], there remains a limited but growing understanding of the transportability of western-developed interventions for use in LMICs (8). A recent systematic review of 13 studies concluded that caregiver-mediated interventions for children with ASD hold promise in India, the second-most populous country in the world (9). The systematic review uses a broad interpretation of the term “caregiver-mediated”, to include a range of models, from parent psychoeducation to intensive centre-based models that combine therapist and caregiver-mediated components, with varying levels of evidence. Sengupta et al., (6) astutely highlighted some ambiguity in the use of the term “caregiver-mediated” within the Indian context, contributing to challenges in interpreting the research evidence in this field [although this is by no means a uniquely Indian challenge; e.g., see (10)]. Of the 13 studies reviewed by Kalorath et al. (9), eight focus on children under 6 years of age (the others include children up to age 9 or 10). Of these, only three report on models that are exclusively caregiver-mediated and relatively resource-efficient (6, 11, 12); others describe programs that involve a therapist-delivered component (13), are longer in duration and/or more intensive [i.e., (14): 19-month model (15), and (16): daily intervention for 3 months], or programs that focus on behaviour reduction (17) or parent psychoeducation (18) rather than direct caregiver coaching to promote the child's developmental progress.

The feasibility of transporting a fully caregiver-mediated intervention for use in LMICs has been demonstrated through a program that was developed and validated in the UK (PACT) (19) and adapted for use in Pakistan and India (PASS) (20). The intervention was delivered to children with autism (aged 2–9 years) over a six-month duration with hour-long sessions every other week (i.e., 12 sessions). A randomized control trial (RCT) with 65 families yielded a high completion rate (81%) and positive outcomes for parental synchrony and child initiations to parents, but not for their secondary outcomes of child language skills. This work shed light on the opportunity to capitalize on effective, resource-efficient intervention approaches for application in LMIC contexts. The authors selected a broad age range for participants due to evidence that age of diagnosis was elevated in south Asia (vs. the UK) at the time of the study. Although approximately half the sample was under age six (mean age ∼5 years), it is not explicit in the paper how many children were in the toddler age range, and children with developmental levels below 12 months were excluded. It remains to be determined whether such an approach would be equally feasible and efficacious in a toddler-aged sample.

Manohar et al. (11) used an individualized approach to supporting parents to deliver caregiver-mediated intervention to young children (aged 2–6 years) with autism. They reported on an RCT design (*n* = 50) evaluating a 12-week NDBI-based model, entailing five, 1:1 outpatient visits to support children's joint attention and adaptive skills (only three sessions were dedicated to the actual intervention training). Following the brief intervention, parents reported reduced stress, and clinician-rated autism symptoms decreased, with a small effect size. These findings are compelling, particularly given the very brief nature of the intervention. However, the lack of a manualized curriculum will pose challenges to any efforts to replicate and to scale up the model. Further work to standardize and manualize this program may be of value given the resource-efficiency and reported outcomes.

Sengupta et al. (6) reported on the implementation of a manualized program (Project ImPACT) for children aged 1–6 years, adapted for use in the Indian context. The program entailed twice-weekly sessions over six weeks, including five direct coaching sessions, five group learning sessions, and two introductory sessions. They reduced the standard (12-week) model to 6 weeks to encourage retention, and reported a high completion rate (85%). A pre-post design revealed that parents learned the intervention techniques and reported significant reductions in parenting stress and gains in children's social communication skills. Authors acknowledge that this study would have been strengthened by the inclusion of measures beyond parent-reported outcomes, but findings nonetheless contribute to the evidence that caregiver-mediated programs hold promise for use in the Indian context; moreover, the potential of training parents remotely has also been demonstrated through a virtual coaching approach in a small sample [*n* = 12; (21)].

In response to calls for caregiver-mediated treatment approaches across the globe, the WHO partnered with Autism Speaks to develop the Caregiver Skills Training program (WHO-CST) (5). Initial development work yielded a framework for designing a globally oriented caregiver-mediated approach for children with a range of developmental challenges across early childhood (age 2 to 9 years). Several key considerations were identified, including engagement of other family members, caregivers' wellbeing, acceptance of their child's learning needs, mitigating barriers to accessing the program, and addressing the variability of children's strengths and needs. The advantage of combining group-based and individual instruction was also highlighted, as a means to balance efficiency, efficacy, and individualized care. Field trials of the WHO-CST program across 30 countries revealed that most sites identified a need for only minor modifications to increase transportability (language, use of idioms) and feasibility [offering childcare, reducing the frequency of group sessions; (5)].

A recent report (12) demonstrated the feasibility of using the WHO-CST program in India with children with social communication challenges, delivered within a school setting. Based on a pre-post design with 22 families, the program (which entailed 9 group sessions and 3 home visits) was found to be acceptable and feasible, and parents reported reduced stress and gains in their own skills and knowledge. Moreover, developmental gains emerged in children's social communication skills (rated by parents and clinicians). Some barriers emerged, related to logistics (e.g., missing work to attend sessions) and time constraints, but the program was felt to be feasible for implementation across a wide range of ages and developmental needs. Again, the focus here was on children from preschool age to school age, with no participants under age three (although the program was available as early as age two).

The bottom line is that, despite some promising evidence, there remains a need for further evaluation of programs that are resource efficient (i.e., exclusively caregiver-mediated, briefer duration, group-based), and that focus on the toddler years, when learning is likely to have the greatest impact (22). Given a growing appreciation for the unique needs of autistic toddlers, it is worth building on the successes of caregiver-mediated programs such as PASS, Project ImPACT, and WHO-CST to examine the transportability of programs that are developed specifically for *toddlers* with emerging autism. The implementation of toddler-focused approaches is becoming increasingly viable with an increasing recognition that ASD can be detected reliably before age two (1, 23), in tandem with evidence to support the urgency of earliest possible intervention (22).

The Social ABCs is a parent-mediated intervention developed in Canada. The program was designed specifically for use with toddlers (aged 12–36 months; with current clinical application up to age 42 months) with autism or related social communication challenges and has been adapted to optimize resource-efficiency. The program's original model entails individual (1:1) sessions for both learning content and live coaching, tapering in intensity over a 12-week period, with evidence of parent and child gains in a cross-site RCT (24) and a large community implementation trial (25). With the aim of increasing efficiencies and optimizing the benefits of group-based learning, the program was adapted to be delivered as a brief, hybrid group-based and individual mode (i.e., 6, weekly group sessions for didactic instruction and facilitated discussion; and 9 sessions of 1:1 in-the-moment coaching), taking place over a 6-week period. In addition to increasing resource efficiency, the truncated duration may increase the program's feasibility for families with multiple competing demands and busy schedules [e.g., see (6)]. Findings from a quasi-experimental, pre-post study of the abbreviated group-based Social ABCs (*n* = 82) showed that parents could learn the strategies in the abbreviated format (i.e., they attained fidelity of implementation). Moreover, toddlers made significant gains in vocal responding (based on blinded video coding), and parents reported increased word inventory and reduced autism symptoms in their toddlers, as well as reduced parenting stress. In-person and virtual delivery of the program yielded very few differences in outcomes, with the exception of parenting stress which was mitigated most strongly in the in-person delivery condition (26).

Due to its resource efficiency (short duration, minimal staffing resources needed), the Social ABCs holds promise for scalability to LMICs. The program also meets many of the criteria proposed by the WHO for such models, including encouraging involvement from other family members (e.g., discussion about how to get other family members involved in supporting the coached parent), a focus on caregivers' wellbeing (e.g., stress, self-efficacy, empowerment), acceptance of the child's learning needs (e.g., increased understanding of toddlers' motivation, arousal, interests, and communication cues), and personalizing specific program goals in response to individual toddlers' and caregivers' strengths and needs. The group-based version of the Social ABCs program holds particular promise as being feasible for delivery in LMICs, by virtue of its efficient (6-week, group-based learning) approach, and opportunity for virtual delivery. Although developed and evaluated to date exclusively in Canada, the group-based Social ABCs has been shown to translate well across diverse ethnic, educational, and language communities within a large multi-cultural city (26), bolstering the promise of this program for implementation outside of Canada.

The primary objectives of the current study were to describe the virtual training partnership and explore preliminary indices of feasibility, acceptability, and efficacy of the 6-week, group-based Social ABCs, when delivered through a clinical service in Goa, India.

## 2 Method

### 2.1 Participants and setting

Clinicians at the Sethu Centre for Child Development and Family Guidance (hereafter Sethu), a child development centre in Goa, India, were trained by the Canadian program-development team (hereafter, “training team”); training details are described below. Sethu runs as a charitable trust (not-for-profit organization), providing services to children with developmental, behavioural, emotional, and learning challenges from birth to age 19 years. Data were extracted from clinical charts at Sethu, for all families who received the Social ABCs intervention between January 2021 and September 2022.

### 2.2 Compliance with ethical standards

#### 2.2.1 Research involving human participants

This study entails an anonymized chart review of clinical data from families enrolled in the Social ABCs as a clinical service. As such, the requirement for consent was waived and the study was approved by Research Ethics committees at Sethu and the University of Toronto.

### 2.3 Procedure

This study is a retrospective chart review. All available data were used for analyses, using a single group pre-post design.

#### 2.3.1 Training of coaches in India

Two clinicians from Sethu were introduced to the Social ABCs program during an introductory two-day in-person workshop hosted by the Indo-Canadian Autism Network (I-CAN 2020) in Hyderabad, India. I-CAN is a collaborative initiative between researchers, clinicians, and autism experts in Canada and India, co-sponsored by the Divi's Foundation for Gifted Children, which sponsored the event in India, and Kids Brain Health Network, Canada, which sponsored the travel of Canadian delegates to the initial meeting and co-supported portions of the ongoing training through a research grant to lead author (JB). Following the workshop, the two clinicians expressed an interest in pursuing formal training, and the program development team determined that the clinicians and the program setting were both a good fit for further training and implementation of the program. Areas of “fit” included practical considerations such as clinicians' English fluency and backgrounds in child development and autism, as well as an alignment with the values underlying the program itself (e.g., child- and family-centred care, family empowerment). Following the initial in-person meeting, the Indian and Canadian teams continued to meet virtually using the Zoom for healthcare platform. Weekly, two-hour meetings took place for six months, with Indian trainees working directly with local families from Sethu, while receiving in-the-moment, live meta-coaching from two Canadian trainers. Trainees’ direct implementation with families (a component of the training phase only), their in-vivo parent-coaching skills, and delivery of didactic content/group facilitation were evaluated using Canadian fidelity measures via video coding.

#### 2.3.2 The intervention

All families participated in the 6-week group-based *Social ABCs* as described in Brian, Solish, et al. (26), with four *a priori* modifications (see program adaptations, below). The program involved 1 baseline session, 6 weekly group didactic sessions and 9 individual coaching sessions, and 2 follow-up/ check-in sessions (see Table 1). The program was delivered through the clinical service at Sethu in Goa, India. A total of 11 group cycles were completed, comprising 2–8 families each, and at least 2 coaches/facilitators. All group cycles were completed between January 2021 and September 2022.

**Table 1:** Program protocol.

	Week 1	Week 2–4	Week 5–7	1 month post-program	3 months post-program
Didactic (group) sessions	–	1/week (total: 3)	1/week (total: 3)		
Coaching (individual) sessions	–	2/week (total: 6)	1/week (total: 3)		
Other	Data collection (Baseline)	–	Data collection (end of week 7)	Check-in^a^	Check-in^a^

^a^ Check-in sessions were added by the clinical team in India; such sessions were not included in previous reports of the group-based Social ABCs program (26).

#### 2.3.3 Program adaptations (*a priori*)

In cross-site planning discussions, we considered the recommended cultural adaptations generated from development of the WHO-CST program, namely: (1) translation into local language, (2) use of examples, idioms, stories that are locally meaningful, and (3) changes to structural elements such as timing and location of sessions, provision of childcare, refreshments, parking, etc. (5). In line with these guidelines, four modifications were made *a priori* to enhance feasibility for families. First, families could opt to be coached in local languages (specifically Hindi, Konkani, or Marathi). Second, the questionnaires (described below) were reviewed verbally with families, in their local languages, as requested. Third, two extra sessions were added following the standard 6-week model. These sessions were provided as follow-up/check-in sessions at 1 and 3 months post-program. These check-in sessions took place virtually or in-clinic, as preferred by families, and each entailed a one-hour consultation/refresher session to provide tips, reminders, or problem-solving strategies to families. Although data were not collected at these follow-up sessions, the primary objective was to encourage parents to continue using the strategies that they had learned, for at least three months following program completion. Thus, the full program entailed 18 sessions, with data collection at the end of week 7 (as depicted in Table 1). The fourth *a priori* modification was that families could access the program either in-person (in clinic), virtually (from their homes), or using a hybrid in-person/virtual format. Decisions about program format were driven by practical factors such as COVID-19 lockdowns at the time, families' network connectivity, and/or the convenience of families coming into the Centre (i.e., not based on clinical impressions about which option would be the best “fit” for each family).

Throughout the partnership, we applied the ecological validity model (5) to consider potential further (*post hoc*) adaptations across eight dimensions: language, persons, metaphors, content, concepts, goals, methods, and context (27).

#### 2.3.4 Data collection and measures

All data were collected as part of the clinical program for quality assurance. Video-recorded parent-child free play interactions and parent questionnaires were collected before starting the program (*Pre*) and following the 6-week intervention (*Post*). The majority of videos were coded by the Indian team, after achieving coding reliability with the training team.

##### 2.3.4.1 Video-coding

In line with established procedures from the Social ABCs research literature (24, 26, 28), ten-minute, parent-child free play interactions were video-recorded at both time-points, with no coaching taking place during the recording. Although video-coding was non-blinded, most videos were consensus-coded in pairs within the Indian team who had established coding consistency with the Canadian team, as follows: *Phase 1*—trainees attended a didactic training session devoted to video coding (and they received a copy of the slide presentation for use as a resource guide for ongoing coding); *phase 2*—weekly supervision sessions during which the trainees in India observed and participated (e.g., providing observations, asking questions) while two members of the program development team coded the videos and explained their codes; *phase 3—*trainees in India scored videos independently and then met bi-weekly with members of the program development team who double-coded the videos with the trainees observing. In those sessions, trainees identified any differences in their coding, and these were discussed and resolved. *Phase 4* entailed trainees independently coding videos and bringing specific coding questions or difficult-to-code videos for discussion during bi-weekly supervision sessions. At the time of writing, the Canadian and Indian teams continue to meet regularly to maintain cross-site consistency.

##### 2.3.4.2 Video-coded variables

Consistent with previous studies (24, 26, 28), video coding examined two primary outcomes, *parent implementation fidelity* and *toddler vocal responsiveness*, as well as a secondary outcome, *toddler vocal initiations.* Continuous interval coding captured parent implementation fidelity. Each 1-minute interval was scored as correct or incorrect/absent for each of ten strategies taught to families [adapted from (29); see (25)]. Total fidelity was the mean percentage of intervals wherein parents demonstrated appropriate use of the techniques [target was ≥75% fidelity, per (30)]. Toddler vocal responsiveness was coded continually and is reported as the percentage of toddler vocalizations directed to the parent in response to parent-provided language opportunities. Toddler vocal initiations were also coded continually and reflect a count per minute of toddler-initiated vocalizations that were directed to the caregiver [see (24)].

##### 2.3.4.3 Parent-report measures

Although the questionnaires were available only in English, all responses from questionnaires were gathered in an interview format with the parents to ensure that they understood each question. This was done in English or in the family's local language as requested, either over the phone or in-person. Questionnaire responses were collected by a clinician who had not coached the family. Data from two parent-report questionnaires were available both before and following the intervention, including a measure of child skills and behaviour and an index of parenting stress. *Child skills/ behaviour*. The Autism Treatment Evaluation Checklist (ATEC) (31) is a 77-item, likert-type checklist (some items are rated on a 3-point scale, others on a 4-point scale), with higher total score indicating greater impairment. The checklist measures child skills/behaviour in areas such as communication, social functioning, sensory, cognitive, health, and physical behaviour, and was developed to measure change associated with treatment. The ATEC is available at no charge and can be scored online, with good internal consistency for use with autistic preschoolers (Cronbach's alpha = 0.91–0.96 for total score). *Parenting stress.* The Autism Parenting Stress Index (A-PSI) (32) is a 13-item, 5-point likert-type scale that is available at no cost through creative commons (http://creativecommons.org/licenses/by-ncnd/3.0). This measure has been validated for use in children with autism under 6 years of age, as a measure of parenting stress that is specific to core and co-occurring autism symptoms. It was designed to measure change in response to intervention. The authors report high internal consistency for use in ASD (Chronbach's alpha = .83) and good test–retest stability [.88; (32)]. Family demographics were accessed through the existing clinical database at Sethu.

### 2.4 Analyses and hypotheses

#### 2.4.1 Feasibility and acceptability

We examined feasibility from the perspective of coaches (did they learn how to deliver the didactic content and coaching through the virtual training model?) as well as by exploring patterns in how families opted to access the program (in-person, virtually, or hybrid; in English or local languages). As an index of acceptability to families, we examined attendance records to explore program completion rates. Program completion was defined as continuing in the program until the end of week 7 (i.e., follow-up sessions were not counted).

#### 2.4.2 Treatment effects

Primary (parent fidelity and toddler responsivity) and secondary (toddler initiations) outcomes were obtained from video-coding of 10-minute toddler-caregiver free play sessions, coded per established methods (see 24, 26, 28). Exploratory outcomes examined changes on the parent questionnaires, and the influence of delivery method and coach on primary outcomes. Paired-samples *t*-tests were conducted using IBM-SPSS Statistics (version 25) to compare pre- vs. post-intervention performance on video-coded variables and parent questionnaires. We hypothesized that we would see gains in both parent fidelity and directed toddler vocalizations following the 6-week training period. No hypotheses were generated for the parent questionnaires, as these were exploratory aims. For significant tests, we report effect size (Cohen's *d*: mean difference/ standard deviation of the difference), interpreted as small (0.20–.49), medium (0.5–.79), or large (0.8 or greater), per Cohen (33). Exploratory analyses used univariate ANOVA to examine differences in primary outcomes across four variables: (1) program delivery method, (2) parent coached, (3) language of coaching, and (4) coach; based on previous reports from the group-based model (26) we did not anticipate any significant differences. Finally, we used Pearson correlations to explore possible associations between outcomes and number of sessions attended, predicting a positive association.

## 3 Results

### 3.1 Normality assumptions

Examination of skewness and kurtosis indicated normality of distributions (i.e., skew ≤ |2|; kurtosis ≤ |3|) for the two primary outcome variables, change in parent fidelity and toddlers' vocal responsivity. The secondary video-coded change variable (toddler initiations) had acceptable skewness, but slightly elevated kurtosis (kurtosis = 3.44). Removal of the top 3 outliers for this variable yielded acceptable kurtosis (1.50); these data points were removed for further analyses.

### 3.2 Sample description

A total of 64 primary caregivers (82.2% mothers) and their toddlers (79.7% boys); mean age = 28.5 months (range 19–35 months) enrolled in the clinical service, across 11 cycles of the group-based program. Groups ranged in size from 2 to 8 caregivers. All toddlers had received a clinical diagnosis of ASD from clinicians at Sethu. Table 2 displays family demographics (e.g., toddler age, birth-assigned sex, caregivers' identified role, education, income, area of residence, home language, number of children in the home, and whether the child is currently in school). The two primary coaches coached over 91% of families, with the remainder being coached by the second round of trainees.

**Table 2:** Toddler and family characteristics and program-related details.

Variable	*N*	Statistics
Toddler age	64	*M* = 28.50 months (*SD* = 4.01) Range: 19–35 months
Sex of toddler (m:f)	64	51:13 (79.7% male)
Number of children in the family	56	One: 38 (59.4%) Two: 16 (25.0%) Three: 2 (3.1%)
Program completion	64	Completed: 54 (84.4%) Did not finish: 10 (15.6%)
Program modality	64	In-person: 19 (29.7%) Virtual: 25 (39.1%) Hybrid: 20 (31.3%)
Caregiver coached	64	Mother: 55 (85.9%) Father: 7 (10.9%) Aunt: 1 (1.6%) Grandmother: 1 (1.6%)
Language of coaching	64	English: 51 (79.7%) Hindi: 5 (7.8%) Konkani: 3 (4.7%) Mixed (includes Marathi): 5 (7.8%)
Educational attainment of coached parent	53	Higher high school credit (equivalent to 12th grade/ high school): 3 (4.7%) Graduate (equivalent to bachelor's degree): 34 (53.1%) Post-graduate (equivalent to master's degree): 16 (25.0%)
Household annual income in Indian Rupees (INR)*	51	INR 5 K—10 K: 3 (4.7%) INR 10 K—19,999: 8 (12.5%) INR 20 K—29,999: 8 (12.5%) INR 30 K—39,999: 4 (6.3%) INR 40 K—49,999: 8 (12.5%) INR 50 K—100 K: 14 (21.9%) >INR 100 K: 6 (9.4%)

* Approximate exchange rate in 2021 for Indian Rupees (INR) to Canadian dollar (CAD) = 1 INR:0.017 CAD (www.bankofcanada.ca).

### 3.3 Feasibility and acceptability

#### 3.3.1 Virtual coach training (feasibility)

After completing the 6-month virtual training, the two primary trainee coaches achieved ≥ 85% fidelity in both implementation and coaching fidelity (based on video coding by senior members of the program development team), each with three different parent-child dyads, and were certified as Social ABCs coaches. Once certified as coaches, both trainees from India advanced to the “Site Trainer” level, allowing them to lead the training and evaluation of two internal staff, supported by the Canadian trainers. The two Indian site trainers are now poised to train other clinical sites in India, thus enhancing the sustainability of the program.

#### 3.3.2 Program delivery mode—setting and language (feasibility)

The program was accessed almost equally across the three delivery modalities: In-person at the centre (*n* = 19; 29.7%), virtual (*n* = 25; 39.1%) and hybrid (*n* = 20; 31.3%). In terms of language of coaching, 13 families opted to be coached in a local language (5 Hindi, 3 Konkani, 5 mixed including Marathi); the remainder were coached in English.

#### 3.3.3 Program completion (acceptability)

**Figure 1:**
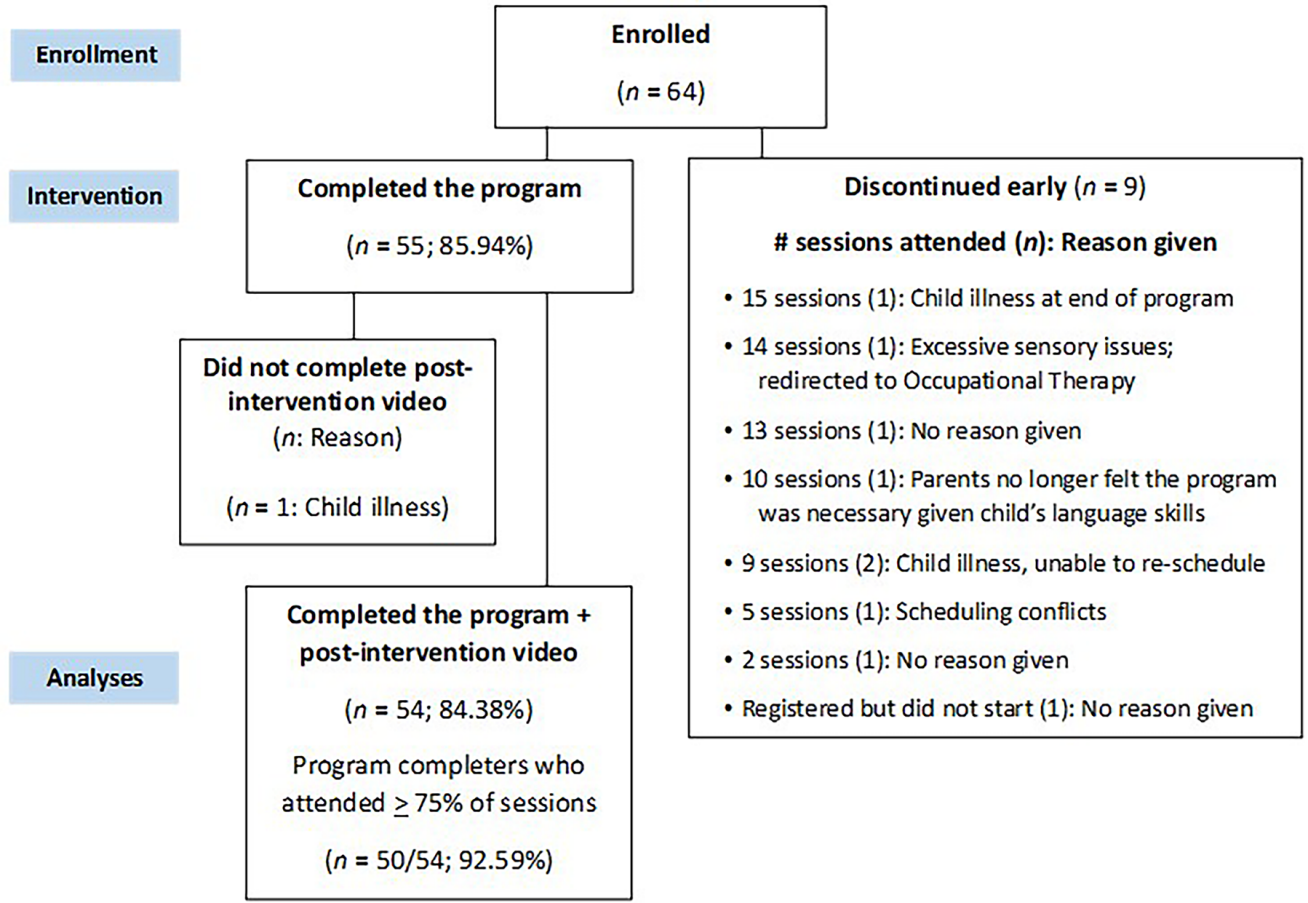
CONSORT diagram of participant flow.

Of the 64 families who enrolled, 55 (85.94%) completed the program; post-intervention video data were available for 54 dyads (see Figure 1 for CONSORT diagram). Of the 9 families who did not complete the program, 3 were due to illness (e.g., COVID; other child health issues), 1 reported scheduling conflicts, 1 child experienced significant sensory challenges and was redirected to occupational therapy, 1 caregiver felt the program was no longer necessary for their child, and 3 gave no reason for discontinuing. Across participants, families accessed an average of 15.47 sessions (*SD* = 3.05). Of the families who completed the program, 50/54 (92.59%) attended ≥ 75% of sessions. While some families attended fewer than 16 sessions, some received more than 16 if sessions were cut short and needed to be rescheduled, or if families had to withdraw from the program and rejoin some time later. Although not required for program completion, 28 families attended at least one of the follow-up sessions.

#### 3.3.4 Program adaptations

Minimal adaptations were made to the program. The most notable were cultural adaptations in the use of *language and metaphors*. Thirteen families were coached in a local language and questionnaire items were delivered in interview format. Early in the process of learning and program delivery, the Indian coaches modified the use of examples and metaphors (which are typically individualised in the Social ABCs) to increase cultural relevance to families in India. As an example, in the original training materials, coaches use the analogy of a multi-layered “banana split” to help families understand the differences between *communication* and *speech* (i.e., the use of a word is the *cherry on top*, that many families seek, but it does not meaningfully stand alone without the foundation of the remaining ingredients). To make the metaphor more culturally meaningful, the coaches in India replaced the “banana split” with a “Gadbad ice-cream”, a local multi-layered ice-cream dessert. Other *a priori* adaptations were structural (e.g., adding follow-up sessions and offering a range of delivery formats).

**Figure 2:**
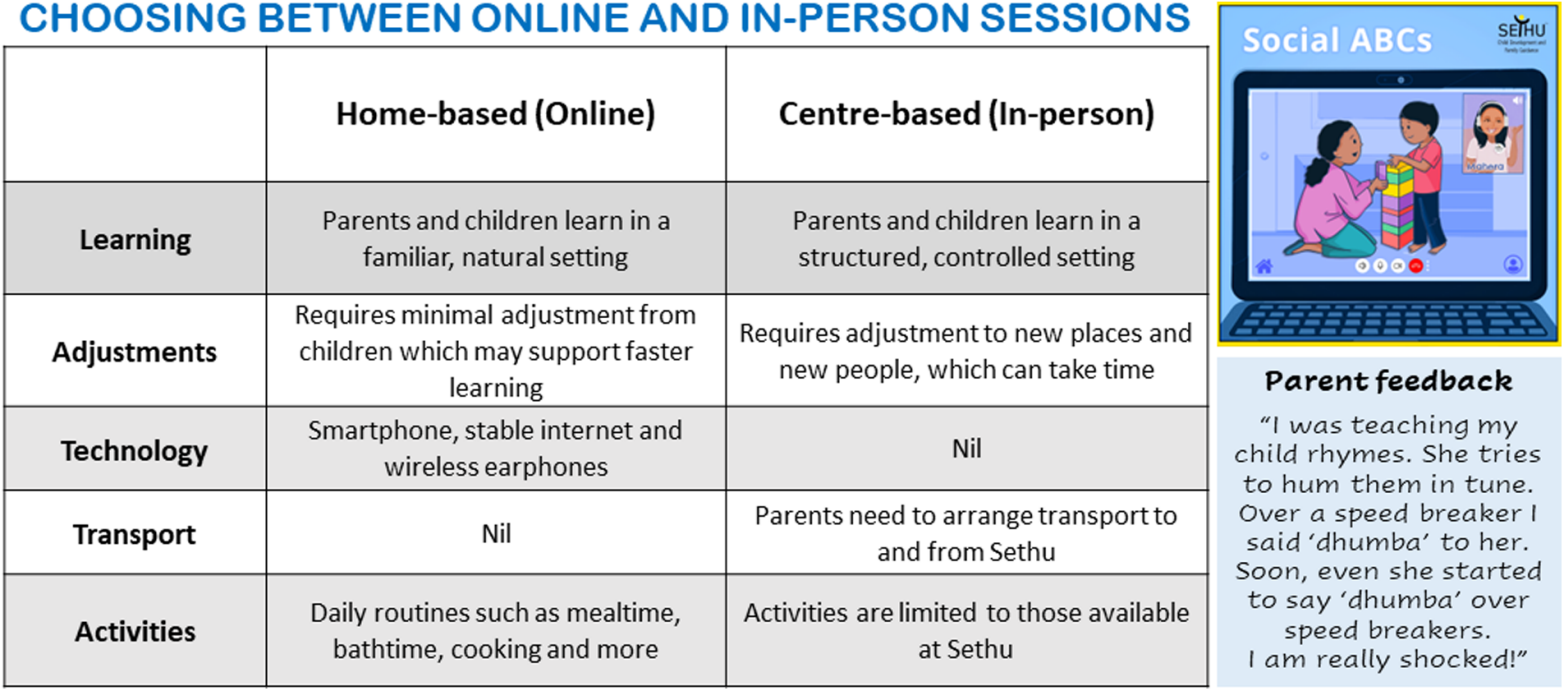
Sample from program advertisement flyer developed at Sethu to help families make program decisions.

Other adaptations were made *post hoc* in the domain of *methods* [as described by (27)]. Innovations made by the Indian team included the development of an introductory information flyer to support parents' understanding of, and navigation into, the program. The flyer outlines the meaning of “parent-mediated”, highlights the program's goals, and delineates the different coaching modalities and their associated considerations to help families distinguish between options (see Figure 2). To support program entry and help families be fully informed, a set of “next steps” for interested families was also developed (i.e., “To know more about the Social ABCs: (1) Join the Parent Support Group, (2) Speak with families that attended the Social ABCs, (3) Visit www.socialabcs.ca, (4) Contact the front desk”). The local team also decided to offer two follow-up sessions as a way to encourage families to keep using the strategies up to three months following the program.

In addition to program modifications described above, one notable adaptation was made to the *training* curriculum. Specifically, the coaches in India expressed the need for mentorship in understanding the unique needs of pre-diagnostic or newly diagnosed toddlers and their families. In response, the Canadian training team provided enhanced learning around “autism literacy” for the coaches in India. We use the term “autism literacy” to refer to an understanding of the unique developmental needs of autistic toddlers and their families (Dowds, personal communication). Concepts include dysregulation, social communication, and attention differences that may be unique to autistic toddlers; the central role of responsive parenting in the context of autistic toddlers providing “cues” that may be difficult for caregivers to interpret; and an enriched appreciation of the impact of a new diagnosis on the family, particularly when engaging families in caregiver-mediated intervention. This enhanced knowledge led to the site leads at Sethu shifting their employment recruiting practices to focus on applicants with education and skills with very young children. Once new trainees were hired, they joined the cross-site meetings where they participated in open-format discussions covering topics such as “toddler development and ASD”, “responsive parenting in autism”, and “supporting families in the early stages of diagnosis”. In addition, the new trainees were required to complete open-access online learning modules prior to starting their training in the Social ABCs, including “Understanding Autism” (34) and “About Autism in Toddlers” (35).

### 3.4 Treatment effects

#### 3.4.1 Video-coded indices

Videos were available at both time points for 48 toddler-caregiver dyads for coding of toddler communication variables, and 47 dyads for coding parent fidelity (seven videos were un-codable due connectivity and/or video quality issues). Data from all dyads with codable pre- and post-program videos were used in the analyses.

##### 3.4.1.1 Parent fidelity

Following the 6-week training period, significant improvements were observed in parent fidelity of implementation from pre- to post-intervention (*M* = 45.42%; *SD* = 11.70 vs. 76.77%; *SD* = 10.74, respectively; 95% CI for the difference = 26.90–35.77; paired samples *t* = 14.21, *p* < .001, Cohen's *d* = 2.07). Over two-thirds of the participants (32/47 = 68.08%) achieved the target of ≥75% fidelity; almost all families (43/47 = 91.49%) achieved ≥ 70% fidelity following the coaching period.

##### 3.4.1.2 Toddler vocalizations directed to caregiver

Significant increases (pre- vs. post-intervention) emerged for child directed vocal responsiveness to parent-provided communication opportunities (*M* = 7.00%; *SD* = 11.14 vs. 46.58%; *SD* = 18.68, respectively; 95% CI for the difference = 34.49–44.67; *t* = 15.65, *p* < .001, Cohen's *d* = 2.26), and rate of child vocal initiations per minute (*M* = 1.16 vs. 3.49; 95% CI for the difference = 1.92 to 4.33; *t* = −5.57, *p* < .001, Cohen's *d* = .83).

#### 3.4.2 Parent questionnaires—child behaviour/skills and parenting stress

Parent questionnaires were available at both time points for only half the sample (*n* = 24). Total score on the ATEC (child behaviour) decreased significantly (*M* = 67.68; *SD* = 19.96 vs. *M* = 47.96; *SD* = 18.93; 95% CI for the difference = −12.99 to −26.44; *t* = −6.06, *p* < .001, Cohen's *d* = 1.21). Mean score on the A-PSI (parenting stress) also appeared to decline (*M* = 13.08; *SD* = 6.85 vs. *M* = 10.32; *SD* = 6.99; *t* = −1.93; 95% CI for the difference = −.19 to 5.71), but this change did not quite reach statistical significance; *p* = .056, and was characterised by a small effect, Cohen's *d* = .38. Post-hoc examination revealed that the study was under-powered to detect a small effect with the current sample size [actual power = 27–39% given *n* = 24, with alpha set to.05; see (33)].

#### 3.4.3 Exploratory analyses

No significant effects emerged regarding the impact on primary outcomes (parent fidelity, toddler responsivity) across program delivery method (*F*(2,44) = .59, *p* = .56 and *F*(2,45) = 1.03, *p* = .36, respectively), parent coached (aunt and grandmother removed for this analysis; *F*(1,44) = .72, *p* = .40 and *F*(1,45) = 2.31, *p* = .14), language of coaching (English vs. other; *F*(1,46) = .24, *p* = .63 and *F*(1,45) = 1.17, *p* = .28), or which coach delivered the program (Coach 1, Coach 2, other; *F*(2,44) = 1.44, *p* = .25 and *F*(2,45) = 1.57, *p* = .22). Finally, no significant associations emerged between number of sessions attended and outcome measures (Pearson *r* = −.005, *p* = .97 for parent fidelity; *r* = .026, *p* = .86 for toddler responsivity).

## 4 Discussion

Study findings demonstrate the feasibility and acceptability of a Canadian-made caregiver-mediated intervention, for toddlers with emerging/confirmed autism, when implemented in Goa, India. Moreover, a pre-post design revealed, based on video-coded free-play sessions, that parents learned how to use the strategies, and toddlers responded and initiated significantly more to their caregivers following the 6-week program. Parents also reported improvements in child behaviour/skills, and a non-significant trend toward reduced parenting stress.

### 4.1 Virtual training of coaches was feasible

Coaches in India were successfully trained, with all formal training occurring virtually. The two primary coaches (Coach 1 and 2) achieved coaching fidelity and went on to be credentialed as Site Trainers, allowing them to train others at their local site with minimal ongoing support from the Canadian training team. In the course of the partnership, the two primary coaches successfully trained two new local clinicians, and they are poised to train other sites in India, with minimal ongoing support. Implementing a train-the-trainer model has been recommended as an effective means of enhancing the sustainability of a program, particularly in a global public health context as it relates to autism care (36).

### 4.2 The program was feasible

Once trained, the coaches were able to deliver the program within their clinical setting in India. Flexibility in language and mode/setting of program delivery may have optimized participation. Approximately 20% of families elected to be coached in a local language other than English, with no difference in outcomes across the language in which coaching occurred. Although the Social ABCs is currently being delivered clinically in languages other than English (i.e., French, Hebrew), this study is the first to report on data from families coached in a non-English language. That there was no difference in parents' fidelity of implementation or in children's responsivity to caregivers, points to the adaptability of the program across cultural contexts and is in line with findings from other caregiver mediated approaches used in India [e.g., (6)]. Approximately 70% of families opted to access at least some of the program remotely (either fully virtual or hybrid), while 30% received the program in-centre. As with coaching language, this element of flexibility did not interfere with program outcomes and supports the power of family choice within the constraints of the core program components.

### 4.3 The program was acceptable

The high retention rate (over 85%) revealed that the program was acceptable to families. Retention rates were remarkably similar to those reported in other studies of caregiver-mediated programs in India [i.e., 85% reported by (4); 81% in (6)] and speaks to both the acceptability of the parent-mediated approach in the Indian context, and to the families' commitment to optimizing available supports for their toddlers with ASD.

### 4.4 The program was efficacious

Caregivers learned the coaching techniques and toddlers made developmental gains. Video-coded outcomes revealed significant increases in parents' use of strategies (fidelity) to support child social communication development and social engagement and significant gains in rate of toddlers' directed vocalizations, both self-initiated and in response to parent-provided language opportunities (responsivity). Although not compared directly, gains were in-line with, or slightly exceeded, those reported in the Canadian context (26) for both parent fidelity (i.e., gains from 35% to 69% and 44% to 71% across in-person and virtual delivery modes, respectively) and toddler responsivity (i.e., from 9% to 55% and 9% to 43% across in-person and virtual delivery conditions, respectively). In addition to gains detected from video-coding, caregivers also reported improved child skills/behaviours on a parent-report questionnaire capturing communication, social functioning, sensory-related and other behaviours often associated with autism.

### 4.5 Trend toward improved parenting stress

A small decrease in parent-reported stress approached, but did not reach, significance. This finding stands in contrast to reports of significant reductions in parenting stress from other caregiver-mediated programs implemented in India (6, 11–13), as well as with published findings from the group-based Social ABCs in the Canadian context (26). While this was a disappointing finding, we were under-powered to detect a true change due to the small sub-sample with available data. We are encouraged by the change in the predicted direction, because the issue of parenting stress, particularly in the context of parent-mediated interventions, is of paramount importance to ensuring parental wellbeing (37).

### 4.6 Program adaptations

The main adaptations were in the areas of language, metaphor, and methods/structure. Specifically, the program was offered in local languages, with customized metaphors, and across three delivery modalities/settings, with two additional follow-up sessions. We were not able to measure the impact of the cultural adaptations, but they were initiated by the Indian team in response to identified caregiver needs and preferences. For example, the Indian coaches developed their own metaphors in response to their front-line experience with families during coaching. The Social ABCs encourages the use of personalised examples and metaphors to support adult learning, even when used in the Canadian context, so it would be difficult to systematically measure their importance here. That ∼20% of families elected to be coached in a local language other than English, raises the possibility that these families might not have been able to access the program without this modification; however, this hypothesis remains untested and warrants closer examination in future work. Finally, the Indian site was one of the first Social ABCs providers to explore offering the Social ABCs using a “hybrid” service delivery model (a mixture of in-person and virtual sessions). The hybrid model was appealing to participating families, with almost one-third opting for this approach, allowing for care that is individualized and family-centred. Moreover, the delivery of the program using a hybrid approach was just as effective as a fully in-person or fully virtual delivery, speaking to the flexibility of the program. The extent to which this modification was context-specific is not clear; indeed several clinical implementation sites in Canada have since been successful in offering a hybrid service delivery model themselves, speaking to the universal appeal of such flexibility.

Aside from language and structural modifications, the necessity of further cultural adaptations was not apparent. This may have been due to existing similarities across cultural contexts, common ground in terms of program values (child- and family-centred care, family empowerment), or the fact that the program was developed within a multi-cultural context (i.e., across two Canadian provinces with varying demographics and cultural identities, including within a large ethnically and culturally diverse city), with Social ABCs coaches having had substantive experience working with diverse families [see (26)]. During the partnership, both teams remained open to further adaptations, as the Indian team came to understand the program and the Canadian team came to learn more about the Indian context. The team did not identify the need for adaptations with respect to the people delivering the coaching, the program content or concepts, goals, or instructional methods (didactic and coaching sessions). Some additional modifications were made by the team in India, but these were not felt to reflect “cultural” differences *per se*. The team's development of novel information fliers reflects a customization of the program that is strongly encouraged for all implementation partners, both within Canada and elsewhere. Although the core components of the program are specified and non-negotiable (e.g., learning content, coaching techniques, parent strategies, targets, manual, didactic materials, session schedule and duration), the program was developed with room for customization to meet each family's unique learning needs and cultural context (e.g., use of examples that are relatable to a particular family, program descriptions, and visuals to help families make meaning of the program).

We do note that a modification was made to the training curriculum, but again, this was not felt to be “culturally” specific and has now been added to training curricula in the Canadian context as well. This included additional learning opportunities around “autism literacy”, to help coaches gain enhanced knowledge and understanding about the unique needs of young autistic children and their families, particular to the toddler developmental period. Areas of focus included emotion regulation and arousal, communication, attention, and the parenting perspective of families in the early stages of learning about a young child's diagnosis while also navigating their own new learning about their child and how best to support them.

## 5 Strengths and limitations

The current study is distinguished by its focus on a resource-efficient (brief, exclusively caregiver-mediated) program, developed for and used with toddlers (all under age 3) with autism. The standard (12-week, individual) program is supported by evidence of efficacy from a cross-site RCT (24), and effectiveness from a large community implementation in Canada (25), and the abbreviated group-based model has been validated in a pre-post design (26). The current paper describes outcomes from the group-based model when delivered through a clinical service in India. *Strengths* include our moderate-sized community sample, flexibility in program delivery modality/setting and language, and video-coded measures in addition to parent-reported outcomes. The two primary coaches were trained virtually, and attained ≥ 85% coaching fidelity. They coached the vast majority of families, and subsequently trained two further coaches who also contributed to the data. *Limitations* include the lack of a control group, lack of blinded video coding and inter-rater reliability checks, and a high rate of missing data for the parent questionnaires. We also acknowledge that our measures of feasibility and acceptability are limited. A richer understanding of caregivers' experiences in the program could have been gathered via parent feedback, which was not available for this study. Finally, the parent-report questionnaires were selected for accessibility in a low-resource setting (i.e., available at no cost), but were not available in the families' local languages. To mitigate this challenge, clinicians administered the questionnaires in an interview format in the language(s) preferred by each family, which may have impacted their standardization. As efforts are being made to increase access to research for communities and researchers in LMICs (38), it will become necessary to improve access to the resources required for research activities (e.g., outcome measures at no cost, with valid cultural and language translation). Despite these limitations, this study demonstrates the feasibility, acceptability, and preliminary efficacy of implementing a brief caregiver-mediated intervention with autistic toddlers in India. Future research would benefit from larger samples, inclusion of a control group (e.g., waitlist controls awaiting the service), blinded and independent video-coding, and standardized questionnaires in local languages. Obtaining detailed caregiver feedback (e.g., satisfaction ratings, qualitative interviews) would lead to a deeper understanding of the factors that make a program more (or less) feasible and acceptable, which should be prioritized in future work.

The 2022 Lancet Commission on the Future of Care and Clinical Research in Autism highlighted the importance of intervening in the first three years of life, noting that “substantial evidence and developmental theory support early initiation of services as soon as signs are observed” (1). Despite concerns that the average age of diagnosis in LMICs exceeds that of western countries (4), the current paper shows that it is possible to identify and initiate intervention with autistic toddlers before age three in India, the second-most populous LMIC in the world. Parent-mediated interventions have potential to empower parents and foster developmental progress for autistic toddlers. Moreover, they may be the most feasible solution for timely intervention in under-resourced, densely populated LMICs, where resources tend to be constrained and scalability is a priority (6) as well as in hard-to-reach communities within high income countries (HICs; e.g., in the Canadian north).

## 6 Conclusions

The current study demonstrated that virtual training can be an effective way to train clinicians to become coaches of a parent-mediated intervention. The program appeared to be acceptable and feasible in the Indian context, caregivers learned the techniques, and toddlers made gains on the video-coded and parent-reported outcome measures. The work reported here was a partnership between the local team in India and the Canadian program developers, aligning with recent calls to avoid “parachute research… where HIC researchers drop into a country, collect data and return home to write a paper without involving or acknowledging the contribution of local researchers or experts” (38). Virtual training of coaches allowed them to practice the program *in situ*, entirely with local families, informing adaptations that were more likely to be meaningful in the Indian context. Sustainability measures were embedded from the start to foster sustainment and growth of the local team with minimal ongoing input from the program development team. Future work should continue to build collaborative models that empower local communities to promote interventions that are feasible, meaningful, and sustainable in the local contexts in which they occur.

## Data Availability

The datasets presented in this article are not readily available because these data were collected from retrospective chart review from a clinical program, and are not publicly available. Requests to access the datasets should be directed to jbrian@hollandbloorview.ca.
